# Automated and Interpretable Detection of Hippocampal Sclerosis in Temporal Lobe Epilepsy: AID‐HS

**DOI:** 10.1002/ana.27089

**Published:** 2024-11-14

**Authors:** Mathilde Ripart, Jordan DeKraker, Maria H. Eriksson, Rory J. Piper, Siby Gopinath, Harilal Parasuram, Jiajie Mo, Marcus Likeman, Georgian Ciobotaru, Philip Sequeiros‐Peggs, Khalid Hamandi, Hua Xie, Nathan T. Cohen, Ting‐Yu Su, Ryuzaburo Kochi, Irene Wang, Gonzalo M. Rojas‐Costa, Marcelo Gálvez, Costanza Parodi, Antonella Riva, Felice D'Arco, Kshitij Mankad, Chris A. Clark, Adrián Valls Carbó, Rafael Toledano, Peter Taylor, Antonio Napolitano, Maria Camilla Rossi‐Espagnet, Anna Willard, Benjamin Sinclair, Joshua Pepper, Stefano Seri, Orrin Devinsky, Heath R. Pardoe, Gavin P. Winston, John S. Duncan, Clarissa L. Yasuda, Lucas Scárdua‐Silva, Lennart Walger, Theodor Rüber, Ali R. Khan, Torsten Baldeweg, Sophie Adler, Konrad Wagstyl, MELD HS study group, Kai Zhang, S M Saiful Bari, James Galea, Venkata Sita Priyanka Illapani, William D. Gaillard, Agustín Ibáñez, Evelyng Faure, Manuel Campos, Mariasavina Severino, Domenico Tortora, Giulia Nobile, Alessandro Consales, Aswin Chari, Martin Tisdall, J. Helen Cross, Callum M. Simpson, Yujiang Wang, Luca De Palma, Alessandro De Benedictis, Lucy Vivash, Terence J. O'Brien, Jane De Tisdi, Marina K.M. Alvim, Fernando Cendes

**Affiliations:** ^1^ UCL Great Ormond Street Institute of Child Health London UK; ^2^ McGill University Montreal Canada; ^3^ The Hospital for Sick Children (SickKids) Toronto Canada; ^4^ Department of Neurosurgery Great Ormond Street Hospital London UK; ^5^ Amrita Advanced Centre for Epilepsy (AACE), Amrita Institute of Medical Sciences Amrita Vishwa Vidyapeetham Kochi Kerala India; ^6^ Beijing Tiantan Hospital Beijing China; ^7^ Bristol Royal Hospital for Children Bristol UK; ^8^ Central Emergency Military Hospital Bucarest Romania; ^9^ University Hospital of Wales Cardiff UK; ^10^ Center for Neuroscience Children's National Hospital US; ^11^ Epilepsy Center, Neurological Institute Cleveland Clinic Cleveland OH USA; ^12^ Biomedical Engineering Case Western Reserve University Cleveland OH USA; ^13^ Advanced Epilepsy Center Clínica las Condes Santiago Chile; ^14^ School of Medicine Finis Terrae University Santiago Chile; ^15^ Department of Neuroradiology IRCCS Istituto Giannina Gaslini, Member of the ERN EpiCARE Genoa Italy; ^16^ IRCCS Istituto Giannina Gaslini, Member of the ERN EpiCARE Genoa Italy; ^17^ Department of Neurosciences Rehabilitation, Ophthalmology, Genetics, Maternal and Child Health, University of Genova Genoa Italy; ^18^ Radiology Department Great Ormond Street Hospital for Children London UK; ^19^ Department of Neurology Epilepsy Program, Ruber International Hospital Madrid Spain; ^20^ School of Computing Newcastle University Newcastle upon Tyne UK; ^21^ Medical Physics Unit Bambino Gesù children's hospital, IRCCS, Member of the ERN EpiCARE Rome Italy; ^22^ Functional and Interventional Neuroradiology Unit Bambino Gesù children's hospital, IRCCS, Member of the ERN EpiCARE Rome Italy; ^23^ Department of Neuroscience The School of Translational Medicine, Monash University Melbourne Australia; ^24^ Department of Neurology Alfred Health Melbourne Australia; ^25^ Birmingham Women's and Children's NHS Foundation Trust Birmingham UK; ^26^ College of Health and Life Sciences Aston University Birmingham UK; ^27^ Department of Neurology New York University Grossman School of Medicine New York New York USA; ^28^ Florey Institute of Neuroscience and Mental Health Melbourne Australia; ^29^ UCL Queen Square Institute of Neurology London UK; ^30^ Department of Medicine, Division of Neurology Queen's University Kingston Canada; ^31^ National Hospital for Neurology and Neurosurgery London UK; ^32^ Department of Neurology UNICAMP University of Campinas Campinas Brazil; ^33^ Brazilian Institute of Neuroscience and Neurotechnology Brazil; ^34^ Department of Neuroradiology University Hospital Bonn Bonn Germany; ^35^ Department of Epileptology University Hospital Bonn Bonn Germany; ^36^ Department of Medical Biophysics Schulich School of Medicine and Dentistry Canada; ^37^ School of Biomedical Engineering & Imaging Sciences King's College London London UK

## Abstract

**Objective:**

Hippocampal sclerosis (HS), the most common pathology associated with temporal lobe epilepsy (TLE), is not always visible on magnetic resonance imaging (MRI), causing surgical delays and reduced postsurgical seizure‐freedom. We developed an open‐source software to characterize and localize HS to aid the presurgical evaluation of children and adults with suspected TLE.

**Methods:**

We included a multicenter cohort of 365 participants (154 HS; 90 disease controls; 121 healthy controls). HippUnfold was used to extract morphological surface‐based features and volumes of the hippocampus from T1‐weighted MRI scans. We characterized pathological hippocampi in patients by comparing them to normative growth charts and analyzing within‐subject feature asymmetries. Feature asymmetry scores were used to train a logistic regression classifier to detect and lateralize HS. The classifier was validated on an independent multicenter cohort of 275 patients with HS and 161 healthy and disease controls.

**Results:**

HS was characterized by decreased volume, thickness, and gyrification alongside increased mean and intrinsic curvature. The classifier detected 90.1% of unilateral HS patients and lateralized lesions in 97.4%. In patients with MRI‐negative histopathologically‐confirmed HS, the classifier detected 79.2% (19/24) and lateralized 91.7% (22/24). The model achieved similar performances on the independent cohort, demonstrating its ability to generalize to new data. Individual patient reports contextualize a patient's hippocampal features in relation to normative growth trajectories, visualise feature asymmetries, and report classifier predictions.

**Interpretation:**

Automated and Interpretable Detection of Hippocampal Sclerosis (AID‐HS) is an open‐source pipeline for detecting and lateralizing HS and outputting clinically‐relevant reports. ANN NEUROL 2025;97:62–75

Hippocampal sclerosis (HS) is the leading cause of drug‐resistant focal epilepsy in adults, and the third most common cause in children.^1^, ^2^ It is amenable to surgical resection, with 76% of individuals achieving freedom from disabling seizures 1 year after surgery.^3^ HS is typically diagnosed using structural magnetic resonance imaging (MRI) and is characterized by atrophy (i.e., volume reduction) of the affected hippocampus on T1‐weighted MRI scans, alongside hippocampal T2/FLAIR hyperintensity.^4^, ^5^ Additionally, the affected hippocampus exhibits morphological abnormalities, including increased curvature of the tail^6^ and a reduction in hippocampal dentations.^7^

Despite its characteristic features, magnetic resonance imaging (MRI) abnormalities can be subtle, and HS accounts for approximately 10% of histopathologically‐confirmed focal epilepsy cases that were missed (i.e. MRI negative) by expert epilepsy neuroradiologists.^8^, ^9^ Critically, individuals with “MRI‐negative” scans have significantly lower rates of postsurgical seizure freedom (45%) compared to those with identified lesions (72%–81%).^10^ Attempts to localize lesions involve additional investigations, such as invasive intracranial electroencephalography (EEG), delaying surgical resection, and placing additional burdens on patients and families.^11^, ^12^ Developing imaging software to localize and characterize subtle cases of HS on presurgical MRI scans would aid early diagnosis, surgical planning and potentially improve postsurgical outcomes.

Previous work using machine‐learning has used neuroanatomical features to both distinguish patients with HS from healthy controls (i.e., detection) and lateralize the side of HS‐associated abnormalities (i.e., lateralization).^13^, ^14^, ^15^, ^16^, ^17^ However, these models have often been trained on small, single‐center datasets.^13^, ^14^, ^15^ or on cohorts comprised of exclusively adult patients.^16^, ^17^ They are therefore unlikely to generalize well to other centers with different patient cohorts (in particular pediatric cohorts), MRI hardware, or scanning protocols. Finally, the models published to date have lacked open‐source code and, therefore, cannot be independently validated.

Developing automated tools that work for children and adults is challenging due to the ongoing maturation of the hippocampus during childhood – notably its increase in volume, folding, asymmetries, and myelination.^18^, ^19^, ^20^ Measuring asymmetries within individuals and comparing features against normative growth charts^21^ are potential solutions to adjust for individual differences and to highlight abnormalities across development.

We created an open‐source software for Automated and Interpretable Detection of Hippocampal Sclerosis (AID‐HS) in patients with epilepsy. We leveraged a large, heterogeneous cohort of adult and pediatric patients from 18 epilepsy centers worldwide. We extracted comprehensive surface‐ and volumetric‐based MRI features of the hippocampus using the open‐source tool HippUnfold and used these features to characterize HS abnormalities. These were compared against normative trajectories, and feature asymmetries were used to automate the detection and lateralization of HS. AID‐HS automatically generates individualized and interpretable reports that are tailored to support the presurgical evaluation of patients with suspected TLE. It is shared as an open‐source software to facilitate its clinical evaluation.

## Materials and Methods

### Cohorts and MRI Processing

#### Inclusion and Exclusion Criteria

##### Main Cohort

**FIGURE 1:**
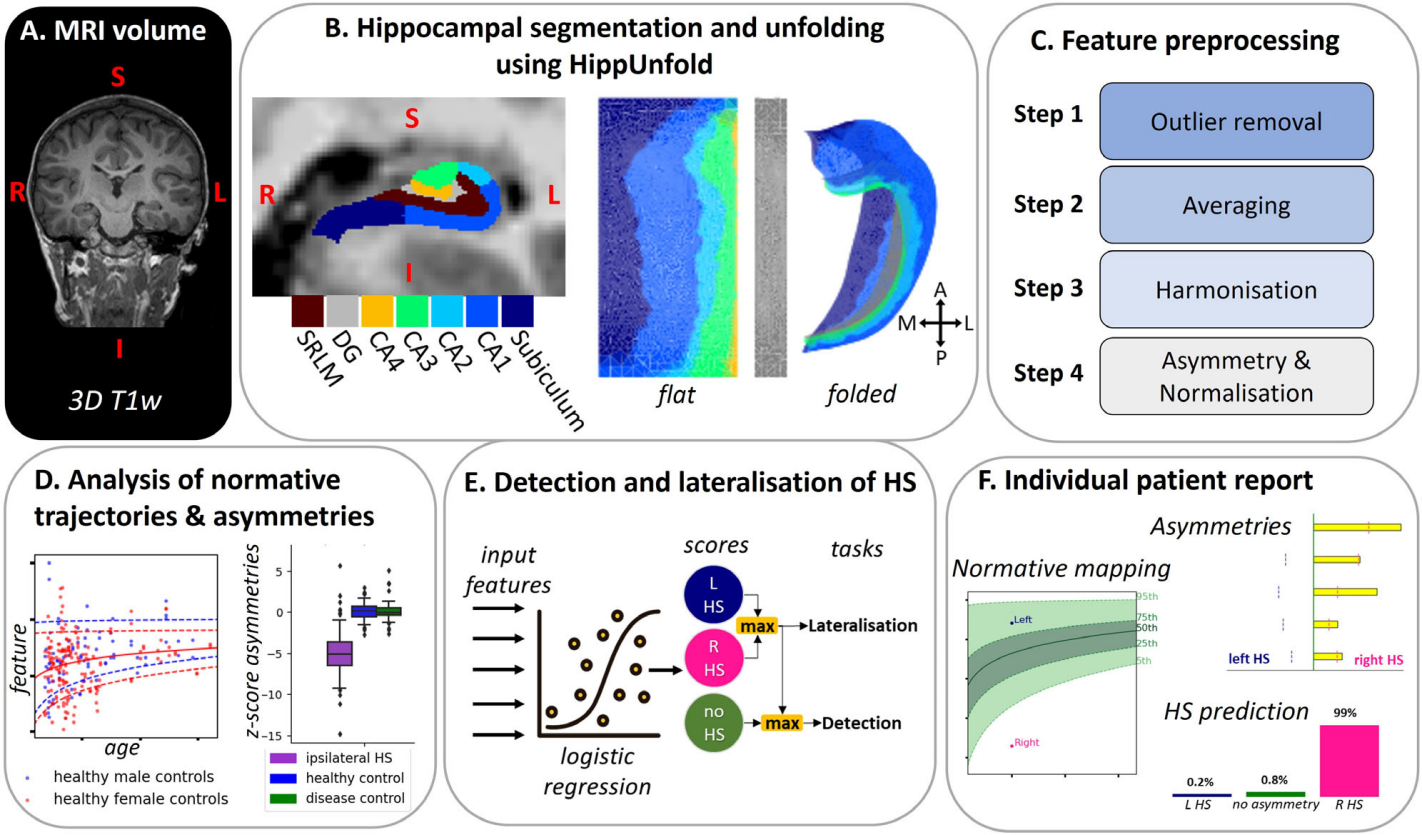
AID‐HS overview. The T1w scan (A) is used as input in HippUnfold, which generates hippocampal segmentations and mesh surfaces that can be visualized flat or folded (B). Surface‐based features undergo preprocessing to remove outliers, adjust for site‐based batch effects, and account for inter and intra‐individual differences (C). Affected hippocampal features are compared to (i) normative developmental trajectories of hippocampal features generated from the healthy controls, and (ii) contralateral hippocampi to characterize the asymmetries (D). Asymmetries are used to train a logistic regression model to predict the likelihood of an individual having a left HS, right HS or no HS. These scores are used to detect and lateralize HS (E). AID‐HS outputs individualized patient reports that detail HS detection and lateralization predictive scores as well as hippocampal feature asymmetries and characterizations of hippocampal abnormalities against normative trajectories (F). AID‐HS, Automated and Interpretable Detection of Hippocampal Sclerosis; CA, cornu ammonis; DG, dentate gyrus; HS, hippocampal sclerosis; SRLM, stratum radiatum, lacunosum, and moleculare. [Color figure can be viewed at www.annalsofneurology.org]

Following UK Health Research Authority approval for the study as well as individual local Institutional Review Board approval from all centers, data were retrospectively collected and anonymized from 4 epilepsy centers: Great Ormond Street Hospital (GOSH), UK; the National Hospital for Neurology and Neurosurgery (NHNN), UK; Beijing Tiantan Hospital (BTH), China; and Cleveland Clinic (CC), USA; prior to sharing with University College London. Informed consent was waived as the analysis was restricted to the reuse of anonymized retrospective data. Patients were included if they had histopathologically‐confirmed HS. Patients reported as having a normal MRI scan at some point during clinical evaluation were considered as “MRI‐negative” patients. Patients who remained MRI‐negative at the point of surgery were lateralized following additional investigations, including positron emission tomography (PET), single‐photon emission computerized tomography (SPECT), video‐telemetry or intracranial EEG. A cohort of patients with focal cortical dysplasia (FCD) were included as disease controls, alongside a healthy control cohort who had been scanned for research purposes. Additionally, 2 patients with reported bilateral HS, diagnosed using semiology, EEG, and MRI scans, were included as independent test examples. Patients and controls were included if they had a preoperative 3D T1w MRI scan acquired at 3T (Fig. 1A) and were more than 3 years old at the time of MRI acquisition. The following clinical variables were extracted: age at MRI scan, sex, histology (ILAE classification where available),^22^ and whether the patients had good postoperative seizure outcome 1 year after surgery (Engel Class 1 or ILAE Class 1).

##### Independent Test Cohort

Data (HS patients, disease controls and healthy controls) from an additional 14 epilepsy centers worldwide were leveraged for independent validation. This included 2 centers where only 1.5T data were available.

### Segmentation of the Hippocampus

MRI data were manually quality controlled. Patients with large imaging artifacts (e.g., motion artifacts impairing the visibility of the anatomical structures) on their preoperative T1w MRI scan were excluded from further analysis.

T1w scans (Fig. 1A) were used as input in the open‐source software HippUnfold ^23^ (Fig. 1B). HippUnfold was developed using healthy hippocampi from young adults from the Human Connectome Project dataset^24^ and validated against manual hippocampal segmentations and other automated hippocampal pipelines.^23^, ^25^ It has previously been used to segment pathological hippocampi in both temporal lobe epilepsy and Alzheimer's disease.^26^, ^27^ HippUnfold uses a U‐Net neural network architecture to segment the hippocampal cortex and applies Laplacian‐based unfolding to fit inner and outer hippocampal surface‐meshes to the T1w images. HippUnfold also provides an automated quality check on the segmentation by examining Dice overlap between the segmentation and a standard template. Following HippUnfold guidelines,^23^ segmentations with a Dice overlap score below 0.7 underwent visual inspection, and subjects with gross segmentation or surface errors were excluded.

For the main cohort, hippocampal volumes were also calculated using FastSurfer,^28^ a deep‐learning accelerated version of FreeSurfer,^29^ which uses a convolutional neural network (CNN) architecture to segment cortical and subcortical structures, including the hippocampus. Total intracranial volume (ICV) was extracted from the FastSurfer segmentation output.

### Extraction of Hippocampal Volume and Surface‐Based Features

We extracted the total volume (in mm^3^) from the hippocampi using both HippUnfold and FastSurfer segmentations. For in‐depth morphological analysis, we used 3 surface‐based features – cortical thickness, gyrification, and curvature – extracted by HippUnfold at every vertex of the hippocampal surface using Connectome Workbench (https://github.com/Washington-University/workbench). Cortical thickness was quantified as the distance between the white matter surface and the pial surface of the hippocampus. Curvature and gyrification measurements were obtained from a surface located at the mid‐thickness between the inner and outer hippocampal surfaces. Mean and intrinsic curvatures were calculated for the outer surface of the hippocampus, and gyrification index was calculated as the ratio between the surface area in its native space and the unfolded space, which measures the degree of surface folding.^30^, ^31^

### Preprocessing of MRI Features

Surface‐based features from the main cohort underwent 4 steps of pre‐processing (Fig. 1C).

Step 1 Outlier removal: Vertices that fell outside 5 standard deviations of the mean distribution were replaced iteratively with the means of their neighbors. Subsequently, surface‐based features were smoothed using a 1 mm full‐width half maximum (FWHM) Gaussian kernel.

Step 2 Averaging: the mean of each surface‐based feature was calculated for each hippocampus (including CA1‐C4, subiculum, and dentate gyrus), excluding 1% of vertices at both extremes of the anterior–posterior axis due to their significant variability.^31^ This produced single mean values per hippocampus for each feature.

Step 3 Harmonization: the averaged features were harmonized using neuroCombat^32^ to adjust for site‐specific biases without removing biological covariates (age, sex, and disease status). The resulting features are henceforth referred to as “harmonized”.

Step 4 Asymmetry and Normalization: asymmetry indexes, referred to as “asymmetries”, were computed to quantify differences between left (lh) and right (rh) hippocampi for all harmonized features following Equation 1. Each subject's asymmetries were z‐scored by the mean and standard deviation across healthy controls to account for typical asymmetry distributions following Equation 2. Output features of this process were referred to as “normalized”.

(1) $${\text{Asymmetries}}_{\mathrm{lh}}=\frac{2\cdot \left({\text{harmonized}}_{\mathrm{lh}}-{\text{harmonized}}_{\mathrm{rh}}\right)}{\left({\text{harmonized}}_{\mathrm{lh}}+{\text{harmonized}}_{\mathrm{rh}}\right)}$$

(2) $$\begin{array}{l} {\text{Normalized Asymmetries}}_{\mathrm{lh}}\\ \;=\frac{{\text{asymmetries}}_{\mathrm{lh}}-\text{mean}\left({\text{asymmetries}}_{\mathrm{lh}}\;\text{controls}\right)}{\mathrm{std}\left({\text{asymmetries}}_{\mathrm{lh}}\;\text{controls}\right)} \end{array}$$

As hippocampal volumes from HippUnfold and FastSurfer were already extracted as averaged hippocampal values, only Steps 3–4 of the preprocessing were applied to them. In the following methods and results, “volume” will refer to HippUnfold's hippocampal volume if not otherwise specified.

On all the data from the independent test cohort, Steps 1, 2, and 4 of the preprocessing were applied to the surface‐based features. However, for data from centers with HS patients and at least 20 healthy controls available, Step 3 of the preprocessing was also applied to further evaluate the impact of the harmonization on the model performances.

### Healthy Hippocampal Anatomy

#### Evaluating HippUnfold in Pediatric and Adult Data Acquired at 3 Tesla

To evaluate HippUnfold performance on pediatric and adult data acquired at 3T, we conducted a comparison between the surface‐based features obtained in our cohort of healthy pediatric controls and adults from the main cohort and those previously computed from a cohort of young adults acquired at 7T from the Human Connectome Project (HCP) dataset, as detailed in DeKraker et al.^23^ To assess the vertex‐level similarities between the 2 cohorts, Pearson's correlation test was conducted, and correlations were corrected for multiple comparisons using the Holm method (alpha = 0.05). A Bland–Altman test was used to confirm this correlation, with limits of agreement set at Mean ± 1.96 SD.^33^

#### Analysis of the Effect of Age, Sex, and Hemisphere on Hippocampal Features in Controls

We used a linear regression model to test the effect of age, sex, and hemisphere on the harmonized features in healthy controls from the main cohort (Table 1).

**TABLE 1:** Demographic and Clinical Information of the Main Cohort and the Independent Test Cohort

Parameter	Main Cohort	Independent Test Cohort
	**HS Patients**	
Participants (n)	154	275
Age at scan, years (median [IQR])	26.5 [18.0‐38.0]	35.5 [27.0‐47.0]
Sex (m:f)	77:77	109:166
Age of seizure onset, years (median [IQR])	9.0 [4.0‐16.0]	10.0 [4.75‐17.0]
MRI‐negative (n, %)	24/154 (15.6%)	14/268 (5.2%)
Good postoperative seizure outcome (Engel Class 1 or ILAE Class 1, 1 year after surgery)	71/99 (71.7%)	167/267 (62.5%)
	**Disease Controls**	
Participants (n)	90	81
Age at scan, years (median [IQR])	14.3 [7.2‐23.0]	15.3 [7.8‐27.5]
Sex (m:f)	46:44	53:28
	**Healthy Controls**	
Participants (n)	121	80
Age at scan, years (median [IQR])	15.2 [11.9‐24.0]	25.5 [13.6‐35.0]
Sex (m:f)	38:83	35:45

Abbreviations: IQR, interquartile range; MRI, magnetic resonance imaging.

### Hippocampal Anatomy in HS

#### Comparison of Features in HS Relative to Normative Growth Charts

Normative growth curves for the harmonized features derived from the healthy controls from the training dataset were generated using Generalized Additive Models (GAMs) accounting for age and sex. Estimates were visualized at the 5^th^, 50^th^, and 95^th^ percentiles of the healthy population. Additionally, percentile scores were calculated for ipsilateral and contralateral hippocampi of each individual in the HS group from the main cohort, as well as for both hippocampi in the disease control group, and compared to the normative growth trajectories (Fig. 1D). GAMs adjusting for ICV were also evaluated, as correcting for ICV in HS morphometry has previously been recommended (Supplementary Figure 2).^34^

#### Statistical Analysis of Hippocampal Asymmetry

In the main cohort, the distribution of normalized asymmetries in ipsilateral hippocampi of patients was compared with hippocampi from both disease controls and healthy controls, where the hemisphere in controls was randomly selected. The normality of each distribution was assessed using the Shapiro–Wilk test. Independent Welch t‐tests (for normally distributed features) and Mann–Whitney tests (for non‐normally distributed features) were conducted to compare the median normalized asymmetries among these 3 groups. P‐values were corrected for multiple comparisons using the Holm method (alpha = 0.05).

A logistic regression was applied to each feature to find the threshold that best distinguishes patients with right/left HS from healthy and disease controls. These abnormality thresholds were further used as a visual guide for reviewing features for individual patients.

### Automated Detection and Lateralization of HS

#### Classifier Training and Leave‐One‐Site‐out Cross‐Validation

Normalized asymmetry features from the main cohort were used to train a logistic regression classifier to detect and lateralize the abnormality. The classifier was set up with a *multinomial* loss, the *lbfgs* solver, and balanced weights to account for multiclass and unbalanced labels. The classifier was trained to classify subjects into 1 of 3 classes – left HS, right HS, or no HS – and generated a score for each of these classes [S_LHS_, S_RHS_, S_noHS_], which sum to 1. These scores were used to assess classifier performance for detection (Equation 3) and lateralization (Equation 4). The classifier was evaluated using leave‐one‐site‐out cross‐validation, meaning that 4 distinct models were trained, each of them trained on 3 sites from the main cohort dataset and tested on the withheld remaining site. This method enables the evaluation of the classifier on the whole main cohort without using the same data for training and evaluation.

(3) $$\text{Clas}s_{\text{detection}}=\left[\text{noHS},\mathrm{HS}\right]*\text{argmax}\;\left(S_{\text{noHS}},\max\left(S_{\mathrm{LHS}},S_{\mathrm{RHS}}\right)\;\right)$$

(4) $$\text{Clas}s_{\text{lateralisation}}=\left[\text{Left}\;\mathrm{HS},\text{Right}\;\mathrm{HS}\right]*\text{argmax}\left(S_{\mathrm{LHS}},S_{\mathrm{RHS}}\right)$$

### Classifier Evaluation on the Main Cohort

Performance was evaluated using 2 tasks: (1) Detection – the ability to distinguish patients with HS from healthy and disease controls accurately, (2) Lateralization – the ability to accurately lateralize the abnormality in patients with HS (Fig. 1E).

To understand the impact of different data on the model performances, the classifier performance on the main cohort was stratified by age (children, adults), sex, histopathology (HS type‐1, HS type‐2, HS type‐3, HS non‐specified), MRI status (MRI‐negative, MRI‐positive), postoperative outcomes, and scan resolution in patients. Classifier performance was also compared between healthy and disease controls. These factors were tested as potential predictors of classifier accuracy using multivariable logistic regression models. Regression coefficients for each factor (β) and their significance (p‐values) were reported alongside the performance breakdowns in Table 3.

### Classifier Comparison with the Baseline Model

Two further logistic regression classifiers with the same parametrization were trained on hippocampal volumes extracted with FastSurfer. One classifier was trained on volumes, while the other was trained on the same volumes after applying Steps 3–4 of the pre‐processing. This comparison allowed the evaluation of the AID‐HS classifier against a volumetric baseline, both with and without pre‐processing techniques. Classifiers' performances were compared in terms of sensitivity at detecting and lateralizing HS.

### Classifier Evaluation on Independent Test Cohort

The 4 models trained on the training datasets were ensembled to form the AID‐HS model. To evaluate the capacity of the AID‐HS model to generalize to unseen data, the model was evaluated on the independent test cohort using the same performance metrics. Performances were assessed separately for scans acquired at 3T and 1.5T (Table 2).

**TABLE 2:** AID‐HS Classifier Performance for the Detection and Lateralization of HS on the Main Cohort and the Independent Test Cohort

Parameter	No. of Subjects	Detection (%)	Lateralization (%)
**Cross‐validation on main cohort**			
Controls	211	94.3%	‐
HS patients	152	90.1%	97.4%
**Independent test 3T**			
Controls	141	95.0%	‐
HS patients	256	89.8%	96.1%
**Independent test 1.5T**			
Controls	20	100%	‐
HS patients	19	89.5%	84.2%

*Note*: Evaluation on the main cohort was performed using leave‐one‐site‐out cross‐validation, where a model was trained on 3 sites and tested on the remaining one, four times to ensure testing on the whole cohort. Evaluation on the independent test cohort is presented separately for data acquired at 3T and data acquired at 1.5T.

Abbreviation: AID‐HS, Automated and Interpretable Detection of Hippocampal Sclerosis; HS, hippocampal sclerosis.

The AID‐HS model was also evaluated on a subset of data from 4 sites where 20 disease or healthy controls were available to perform harmonization (Step 3 of the preprocessing). This test was done to ensure the model performed similarly with and without the harmonization step (Supplementary Table 1).

### Individual, Interpretable Hippocampal Report

AID‐HS generates individualized reports to provide an interpretable characterization of hippocampal abnormalities (Fig. 1F). The reports display HippUnfold segmentations and surface reconstructions alongside automated quality control scores. The segmentation of any hippocampus with a segmentation score below 0.7 must be visually inspected to check whether the segmentation has failed. Left and right hippocampal features are mapped against normative growth charts. Feature asymmetries are displayed to indicate the magnitude and direction of asymmetries, and are compared to abnormality thresholds. Finally, reports include the detection and lateralization scores from the AID‐HS classifier. AID‐HS reports have been co‐designed with the neuroradiologists at GOSH to ensure they meet the clinical needs of providing transparent and comprehensive information that can aid in the diagnosis of patients with suspected HS.

## Results

### Cohort

A total of 815 subjects were initially included in the study: 426 patients with unilateral HS, 2 patients with bilateral HS, 172 disease controls with FCD, and 215 healthy controls. After quality control, 1 participant was excluded due to motion artifacts on their T1w scan, and 12 participants failed the quality check on the HippUnfold segmentation. Table 1 details the final study cohort, split into the main cohort and the independent test cohort.

The main cohort consisted of 365 participants from 4 epilepsy centers: 152 unilateral HS patients, 2 bilateral HS patients, 90 disease controls, and 121 healthy controls. The cohort included 200 adults (≥18 years old) and 165 children (<18 years old), with a median age of 26.5 years (IQR = 18.0–38.0) for patients, 14.3 years (IQR = 7.2–23.0) for disease controls, and 15.2 years (IQR = 11.9–24.0) for healthy controls. There was an association between sex and disease status (χ^2^(2, N = 365) = 11.9, *p* = 0.007). The distribution of males and females was homogeneous in patients and disease controls (Table 1), while approximately two‐thirds of the healthy controls were females. In HS patients, the median age of epilepsy onset was 9.0 years old, and 15.6% of patients were categorized as MRI‐negative. Out of the patients with post‐operative outcomes available (n = 99), 71.9% of patients achieved good postoperative seizure freedom (Engel Class 1 or ILAE Class 1) 1 year after surgery. Among the unilateral HS patients, the breakdown of histopathology diagnoses was 60 HS type‐1, 29 HS type‐2, 7 HS type‐3, and 56 patients with non‐specified HS (e.g. where the histological subtype was not specified in the pathology report). The 2 bilateral HS patients were withheld for testing and therefore were solely included for AID‐HS evaluation.

The final independent test cohort consisted of 436 participants from 14 epilepsy centers: 256 HS patients with scans acquired at 3T and 19 acquired at 1.5T, 141 disease controls and healthy controls acquired at 3T and 20 acquired at 1.5T. Details of the demographic and clinical information for this cohort can be found in Table 1.

All participants had a T1‐weighted whole‐brain MRI scan. In‐plane resolution ranged from 0.39 to 1.19 mm and slice spacing ranged from 0.41 to 3mm. 64.5% of participants had an isotropic T1w (Supplementary Table 2).

### Healthy Hippocampal Anatomy

**FIGURE 2:**
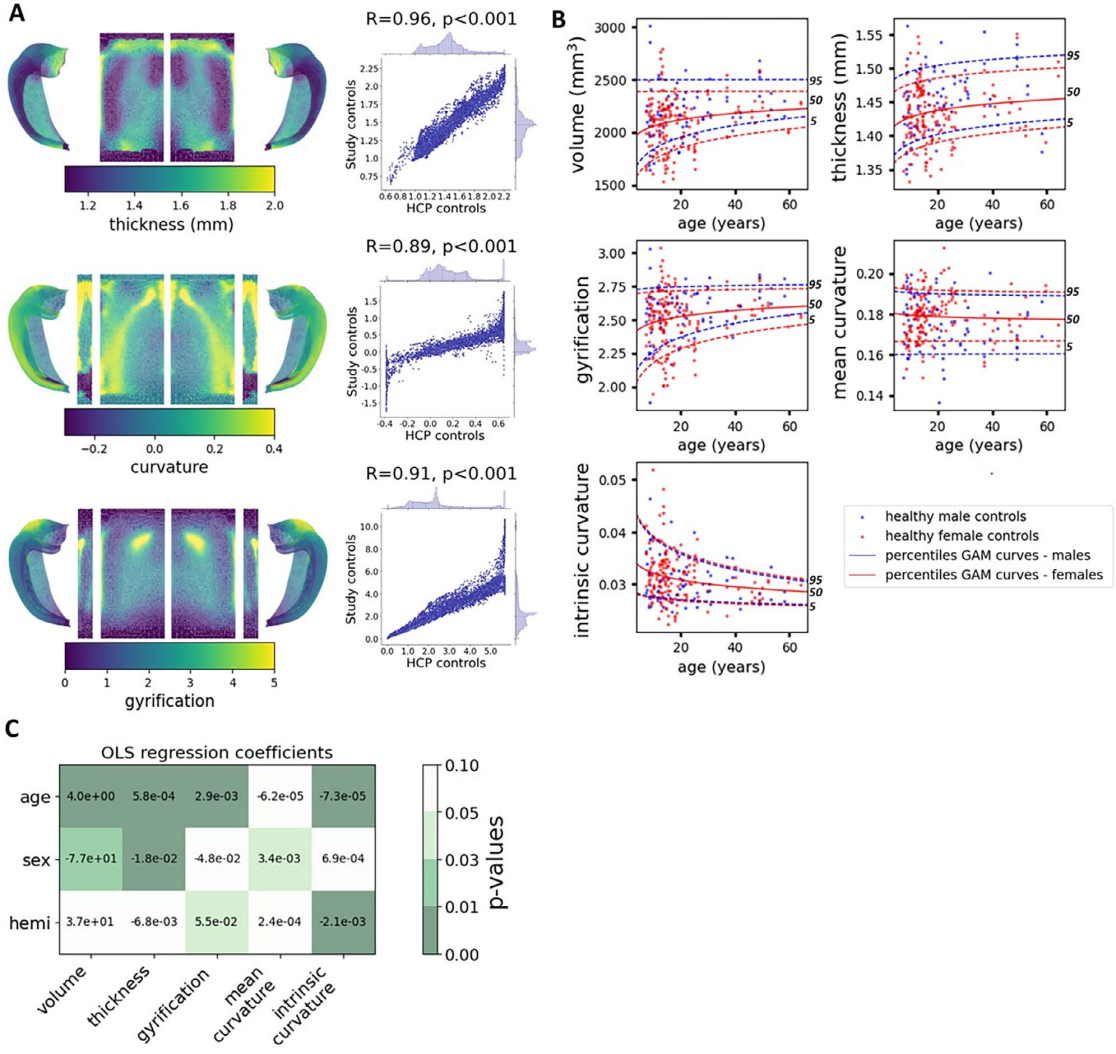
Healthy hippocampal anatomy. (A) Folded and flat maps of HippUnfold‐derived surface‐based thickness, curvature and gyrification in our study's healthy controls. Correlation of surface‐based features extracted from our study's healthy controls compared to the HCP cohort. (B) Normative growth charts of harmonized features in healthy male and female hippocampi for the 5^th^, 50^th^ and 95^th^ percentiles of the population. (C) Coefficients from the linear regression model testing the effect of hemisphere, sex and age on the harmonized features and colored by their significance (*p‐values*). HCP, Human Connectome Project; GAM, generalized additive model; OLS, ordinary least squares. [Color figure can be viewed at www.annalsofneurology.org]

Surface‐based thickness, gyrification, and curvature features from the healthy controls exhibited a similar pattern to those obtained from the HCP young adult 7T cohort (all *R* > 0.89, all *p‐values* <0.001, Fig. 2A). These results were confirmed by the Bland–Altman test which showed no systematic mean differences between features extracted at 3T and 7T, with at least 95% of all points falling within +/− 1.96 SD (Supplementary Figure 1), demonstrating the consistency of HippUnfold performance on 3T MRI. Developmental trajectories of harmonized features in healthy males and females are displayed in Fig. 2B. Except for curvature, all features exhibited significant associations with age (all *p‐values* <0.05). Additionally, volume, thickness and mean curvature were significantly different between males and females, and gyrification and intrinsic curvature were significantly different between the left and right hemispheres (all *p‐values* <0.05) (Fig. 2C).

### Hippocampal Anatomy in HS

**FIGURE 3:**
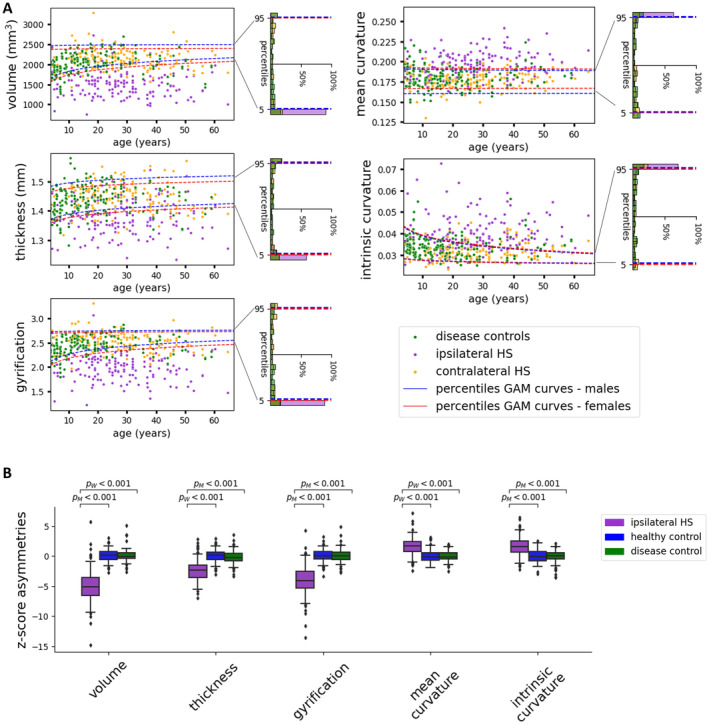
Characterization of morphological abnormalities in HS. (A) Distribution of harmonized features in the ipsilateral hippocampi of HS patients (purple), contralateral hippocampi of HS patients (orange), and hippocampi of disease controls (green) plotted against normative trajectories derived from the 5^th^ and 95^th^ percentiles of the healthy male (blue dashed line) and female (red dashed line) controls' hippocampi. Histograms of the percentage of each group falling within each centile of normative curves are reported on the right axis. Features from ipsilateral HS consistently fell outside of the 5^th^/95^th^ centiles for all features. (B) Boxplots of z‐score asymmetries in ipsilateral hippocampi of HS patients compared to healthy controls and disease controls. Statistically significant differences in distributions between each group were assessed using the Welch T‐test (p^W^) for normal distributions and the Mann–Whitney test (p^M^) for non‐normal ones. HS, hippocampal sclerosis; GAM, generalized additive model. [Color figure can be viewed at www.annalsofneurology.org]

Hippocampal volume and morphological features derived from ipsilateral hippocampi in HS patients deviated from normative curves in healthy controls (Fig. 3A). The hippocampal volume, thickness and gyrification of pathological hippocampi fell below the 5^th^ percentile range of the healthy population for 90.1%, 63.2% and 88.2% of the HS patients, respectively. Additionally, curvature and intrinsic curvature values exceeded the 95^th^ percentile in 67.1% and 70.4% of patients, respectively. Adjusting for ICV showed no systematic improvement in HS centile estimates and was therefore not included (Supplementary Figure 2).

In the analysis of asymmetries (Fig. 3B), patients exhibited significantly more extreme asymmetry values (all *p‐values* <0.001) between their hippocampi in all features compared to both healthy and disease controls. Ipsilateral hippocampi had reduced volume, thickness, and gyrification, and increased curvature and intrinsic curvature in comparison to contralateral hippocampi. No significant differences were observed between healthy and disease‐control groups.

### Automated Detection and Lateralization of HS

On the main cohort, the AID‐HS classifier achieved 90.1% sensitivity in detecting HS and 97.4% in lateralizing HS, with 94.3% specificity in controls (Table 2). In a subset of 24 MRI‐negative patients, the classifier detected 19 patients as being pathological (sensitivity 79.2%) and correctly lateralized 22 patients (sensitivity 91.7%) (Table 3). Among the 12 healthy and disease controls misclassified, 9 were predicted as left HS and 3 as right HS. Among the 10 left HS misclassified, 8 were predicted as having no HS and 2 were incorrectly lateralized as right HS. The 7 right HS misclassified were predicted as having no HS. Classifier performances were not significantly different between adults and children, and between males and females. There was no significant difference in performance between (1) the HS histopathological subtypes or (2) patients who did or did not achieve good postoperative seizure freedom (Engel Class 1 or ILAE Class 1) 1 year after surgery (all p‐values >0.05, Table 3). There was no significant difference in performance based on scan resolution.

**TABLE 3:** Breakdown of AID‐HS Classifier Performance on the Main Cohort

Parameter		No. of Subjects	Detection (%)	Impact of Factor on Detection (β; *p*‐Value)	Lateralization (%)	Impact of Factor on Lateralization (β; *p*‐Value)
**Controls**		**211**	**94.3%**	‐	‐	‐
Group	Healthy controls	121	95.9%	ref	‐	‐
	Disease controls	90	92.2%	β=‐0.67; *p*=0.27	‐	‐
**HS patients**		**152**	**90.1%**	‐	**97.4%**	‐
Age	Adult	118	91.5%	ref	98.3%	ref
	Pediatric	32	85.3%	β=‐0.36; *p*=0.68	94.1%	β=‐0.11; *p*=0.94
Sex	Male	77	90.9%	ref	97.4%	ref
	Female	75	89.3%	β=‐0.03; *p*=0.97	97.3%	β=0.16; *p*=0.89
MRI status	Positive	128	92.2%	ref	98.4%	ref
	Negative	24	79.2%	β=‐1.59; *p*=0.07	91.7%	β=‐1.37; *p*=0.28
Histo‐ pathology	HS type‐1	60	91.7%	ref	100%	ref
	HS type‐2	29	93.1%	β=‐0.13; *p*=0.90	89.7%	β=‐30.4; *p*=1.0
	HS type‐3	7	57.1%	β=‐1.52; *p*=0.14	85.7 %	β=−‐29.4; *p*=1.0
	non specified	56	91.1%	β=25.1; *p*=1.0	100%	β=‐1.6; *p*=1.0
Good post‐ operative seizure outcome (Engel Class 1 or ILAE Class 1)	No	14	85.7%	ref	100%	ref
	Yes	71	87.3%	β=‐0.53; *p*=0.42	97.2%	β=‐1.23; *p*=0.33
Isotropic scan	Yes	94	90.4%	ref	95.7%	ref
	No	58	89.7%	β=0.33; *p*=0.87	100%	β=10.8; *p*=1.00

*Note*: A breakdown of the performance is provided according to age, sex, MRI status, histopathology, seizure freedom, and scan resolutions. Multivariate logistic regression models were used to assess the impact of each factor (*β* coefficients) and the significance (*p‐*values) on classifier performances at HS detection and lateralization.

Abbreviation: AID‐HS, Automated and Interpretable Detection of Hippocampal Sclerosis; HS, hippocampal sclerosis; MRI, magnetic resonance imaging.

In comparison to the AID‐HS model trained on HippUnfold‐derived features, other classifiers trained solely on volumes extracted from FastSurfer achieved 89.8%–90.9% sensitivity in HS detection and 95.4%–94.7% in HS lateralization when using raw volumes and incorporating pre‐processing techniques (Table 4).

**TABLE 4:** Comparison of Detection and Lateralization Performances across Models

Models	Controls and HS Patients Accurately Detected (n, %) N = 363	HS Patients Accurately Lateralized (n, %) N = 152
FastSurfer volumes	326 (89.8%)	145 (95.4%)
FastSurfer volumes + preprocessing	330 (90.9%)	144 (94.7%)
**HippUnfold features + preprocessing (AID‐HS)**	**336 (92.6%)**	**148 (97.4%)**

*Note*: The best model is highlighted in bold.

When evaluated on the independent test cohort with 3T scans, AID‐HS achieved 89.8% sensitivity in detecting and 96.1% sensitivity in lateralizing HS patients, while maintaining 95.0% specificity in controls (Table 2). Moreover, classifier performance was not significantly different when applied to a subset of the independent test cohort processed with and without the harmonization step (Supplementary Table 1), demonstrating that AID‐HS can be used with and without harmonization. Nevertheless, as harmonization is still important for comparison with the normative growth curves presented in the individual reports, the AID‐HS pipeline offers the harmonization as an optional step. This requires a minimum of 20 participants scanned on the same MRI scanner with the same T1w protocol to establish the harmonization parameters.

Finally, AID‐HS achieved 89.5% and 84.2% sensitivity in detecting and lateralizing HS patients and 100% specificity in controls from scans acquired at 1.5T. These results demonstrate the ability of the model to generalize to new unseen scans from different scanners and acquired with different settings, including a lower resolution at 1.5T.

### Hippocampal Reports

**FIGURE 4:**
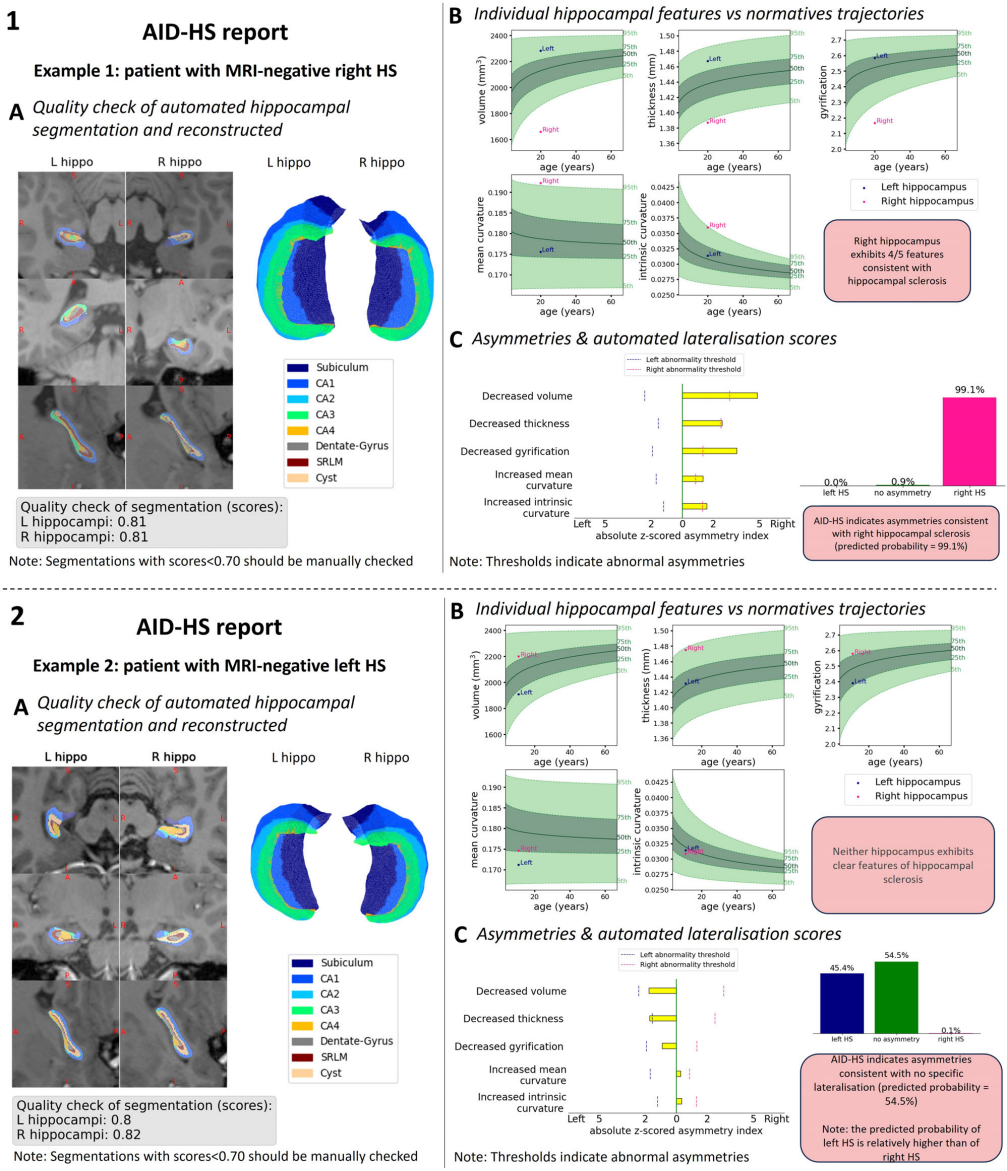
Examples of AID‐HS reports for 2 patients with MRI‐negative right HS (example 1) and left HS (example 2). (A) Automated hippocampal segmentation and reconstructed hippocampal surfaces using HippUnfold, alongside automated quality control of the segmentation. (B) Individual hippocampal features compared to normative trajectories (with 25th – 75th percentiles in dark green, 5th – 25th and 75th – 95th percentiles in light green, patient's left hippocampus in blue and patient's right hippocampus in pink). (C) Asymmetry scores against left and right abnormality thresholds and automated lateralization scores from the AID‐HS classifier, indicating the probability that hippocampal feature asymmetries are consistent with left or right HS or that there is no asymmetry. AID‐HS, Automated and Interpretable Detection of Hippocampal Sclerosis; CA, cornu Ammonis; HS, hippocampal sclerosis; MRI, magnetic resonance imaging; SRLM, stratum radiatum, lacunosum, and moleculare. [Color figure can be viewed at www.annalsofneurology.org]

In each individual, we used hippocampal feature values compared to normative growth charts, hippocampal feature asymmetry scores and results from the AID‐HS classifier to create individual patient reports. Fig. 4 illustrates 2 example reports for patients who were initially reported as MRI‐negative and for whom the HS was lateralized to the right (Example 1) and left (Example 2) side through invasive intracranial EEG, with post‐surgical histopathological confirmation.

In Example 1, automated quality control scores of 0.81 for both hippocampi, indicated good quality hippocampal segmentations (Panel A). Compared with the normative growth charts, the left hippocampus features fell within the normal range of the healthy population, while the right hippocampus had features falling outside the 5th and 95th percentiles (Panel B). In the asymmetry analysis, abnormalities were lateralized to the right hippocampus, with significant reductions in volume, thickness and gyrification, alongside increased mean and intrinsic curvature (Panel C). These findings were further supported by the automated classifier results, predicting right HS with 99.1% probability.

In Example 2, both hippocampi had features within the 5th and 95th percentiles of the normal population. The analysis of the asymmetries demonstrated mixed results, with decreased volume, thickness and gyrification of the left hippocampus consistent with left HS, but increased mean and intrinsic curvature in right hippocampus, consistent with right HS. The classifier classified this patient as having no overall asymmetry (predicted probability of 54.5%), but a higher probability for left HS (45.4%) compared to right HS (0.1%). This report is an example of a more complex patient, with normal hippocampi for the age and head size, and classified as having non‐lateralizing asymmetries. Nevertheless, the in‐depth characterization showed subtle atrophic, thickness and gyrification asymmetries consistent with left HS, which was supported by the higher lateralization score for left HS. This illustrates how the interpretable reports could help inform clinical decision‐making in difficult‐to‐diagnose patients.

AID‐HS was further evaluated on 2 patients with semiology, EEG, and imaging features consistent with bilateral HS (Supplementary Figure 3).

## Discussion

AID‐HS is an automated and interpretable pipeline for the detection and lateralization of HS from T1w MRI scans. We leveraged the open‐source software HippUnfold to extract surface‐based features and volumes of the hippocampus in a large multi‐center cohort of adults and children. We characterized the dynamic development of hippocampal morphology across a wide age range of healthy controls (7 to 60 years old). Our analysis of morphological asymmetry in HS patients revealed significant differences in the pathological hippocampi – characterized by reduced volume, thickness and density of gyrification alongside increased mean and intrinsic curvatures – compared to both healthy controls and patients with focal cortical dysplasia (FCD). These distinctive features were used for automated detection (sensitivity: 90.1%) and lateralization (sensitivity: 97.4%) of HS. Notably, AID‐HS successfully detected and lateralized lesions in the majority of MRI‐negative HS cases (sensitivity: 79.2% and 91.7%, respectively), indicating its potential utility to improve diagnosis in a clinical setting. AID‐HS generates individualized reports that characterize hippocampal abnormalities and provide predictive scores for automated detection and lateralization of HS. AID‐HS is released as an open‐source tool for the epilepsy community.

We extend beyond past studies in several key respects. Compared to previous studies often limited by small single‐center^13^, ^14^, ^15^ or solely adult cohorts,^16^, ^17^ our machine‐learning classifier, trained and validated on 2 different large, heterogeneous multi‐center cohorts, demonstrated consistent performances across a wide range of ages, histopathology subtypes, and MRI scanners. By developing a classifier using heterogeneous data from the most commonly acquired MRI protocol (T1w), we maximize the utility of AID‐HS for epilepsy centers around the world.

Moreover, AID‐HS classifier's performance (90.1% detection and 97.4% lateralization) exceeded that of previous HS classifiers, such as the classifier trained on the large multi‐center ENIGMA cohort of MRI‐positive HS (75% detection and 83% lateralization)^16^ or a previous HS tool for surface‐based features (lateralization only, 93%).^17^ Furthermore, among patients with MRI‐negative HS, our classifier correctly lateralized 92% of the abnormalities, compared to 84%.^17^

Critically, AID‐HS has been shown to differentiate patients with HS from patients with FCD, the most common cause of MRI‐negative focal epilepsy. This is invaluable for the presurgical planning of patients with suspected focal epilepsy with ostensibly normal MRI scans, as the underlying pathology may not be clear a priori.

AID‐HS additionally generates interpretable and individualized reports designed to aid neuroradiological evaluation of difficult to diagnose HS cases.^35^ In this study, we presented 4 examples of diagnostically challenging cases, 2 with HS that had previously been missed on radiological review in which AID‐HS correctly lateralized the abnormality, and 2 cases with bilateral HS which were highlighted by the in‐depth morphological characterization. Moreover, by openly sharing our code and packaging it into a user‐friendly pipeline, we aim to support reproducibility and independent validation of AID‐HS.

Finally, beyond the characterization and detection of HS, this study used the open‐source tool HippUnfold on 3T MRI scans acquired with clinical protocols. Indeed, HippUnfold was used to characterize both healthy development and pathological morphological changes throughout the lifespan. The validation of HippUnfold on clinically acquired data supports future research across fields, ranging from the characterization of longitudinal structural change in epilepsy patients with HS to the study of hippocampal pathology in other neurological diseases, such as developmental amnesia or Alzheimer's disease.

### Limitations and Future Work

A strength of AID‐HS is that the only requirement is a 3D T1‐weighted MRI and the age and sex of the patient. The algorithm was trained and validated on isotropic and anisotropic 3D T1w scans acquired at 3T. However, results from the small subset of patients from the independent test cohort who had 1.5T T1w MRI data demonstrate its ability to generalize to lower‐resolution scans. Nevertheless, further work is needed to fully evaluate the impact of scanner resolution and scan quality on performance and establish the added benefit of additional MRI modalities.

A limitation of AID‐HS is that it is unable to automatically detect bilateral HS or subtype HS. Although we present AID‐HS reports for 2 patients with bilateral HS, the scarcity of bilateral hippocampal cases in our dataset prevented the training of an automated classifier to detect bilateral HS. Nevertheless, in these 2 cases, the interpretable reports clearly characterized the bilateral nature of the abnormalities. The unbalanced distribution of the different HS subtypes (60 HS type‐1, 29 HS type‐2, 7 HS type‐3) and the small number of HS type‐3 prohibited the extension of the model to differentiate subtypes. Further work with a larger cohort, including bilateral cases and HS subtypes, could enable the extension of AID‐HS to automate the detection of bilateral HS and offer HS subtyping.

This is a retrospective study, and future prospective work will be required to assess the added benefit of incorporating AID‐HS in the presurgical evaluation of patients with suspected hippocampal sclerosis. Last, HS can be an incidental finding and AID‐HS can produce false positives. Hence, there is always a need to correlate MRI‐defined HS with clinical and EEG data.

## Conclusion

Our study introduces AID‐HS, an open‐source software for characterizing individual hippocampal morphological abnormalities and automating the detection and lateralization of HS in patients with temporal lobe epilepsy. By utilizing features extracted from commonly acquired T1w scans in a large and heterogeneous cohort of pediatric and adult patients from multiple epilepsy centers, we have demonstrated generalizable performance across a variety of individuals and sites. To facilitate independent evaluation of our software, we have made all the code available on GitHub (https://github.com/MELDproject/AID-HS).

## Author Contributions

M.R., S.A., and K.W. contributed to the conception and design of the study; J.D.K., M.H.E., R.J.P., S.G., H.P., J.M., M.L., G.C., P.S.P., K.H., H.X., N.T.C., T.Y.S., R.K., I.W., G.M.R.C., M.G., C.P., A.R., F.D.A., K.M., C.A.C., A.V.C., R.T., P.T., A.N., M.C.R.E., A.W., B.S., J.P., S.S., O.D., H.R.P., G.P.W., J.S.D., C.L.Y., L.S.S., L.W., T.R., A.R.K., T.B., and MELD HS study group contributed to the acquisition and analysis of data; M.R., S.A., and K.W. contributed to drafting the text or preparing the figures.

## Potential Conflicts of Interests

Nothing to report.

## Supporting information


**Data S1:** Supporting Information.

**Data S2:** MELD HS Study Group.

## Data Availability

All data analysis in this study was conducted using Python. The AID‐HS software is openly available to download (https://github.com/MELDProject/AID-HS).
