# Supplementary material for: Automated and Interpretable Detection of Hippocampal Sclerosis in Temporal Lobe Epilepsy: AID‐HS
Source: Ann Neurol. 2024 Nov 14;97(1):62–75. doi: 10.1002/ana.27089 (PMC11683179; doi:10.1002/ana.27089)
Supplement: Supplementary file 1 — Data S1: Supporting Information. [file ANA-97-62-s001.docx]

**Supplementary material**

**Supplementary Figure 1: Bland-Altman test on features extracted at 3T and 7T.** For every vertex, the difference between the mean feature value extracted at 3T (from the controls in our study) and the mean feature value extracted at 7T (from the Human Connectome Project (HCP)) is plotted against the mean of the 3T and 7T values. Each feature exhibits no evidence of systematic bias (i.e. mean shift ~=0). 95% of vertices fall within +/- 1.96SD from the mean.


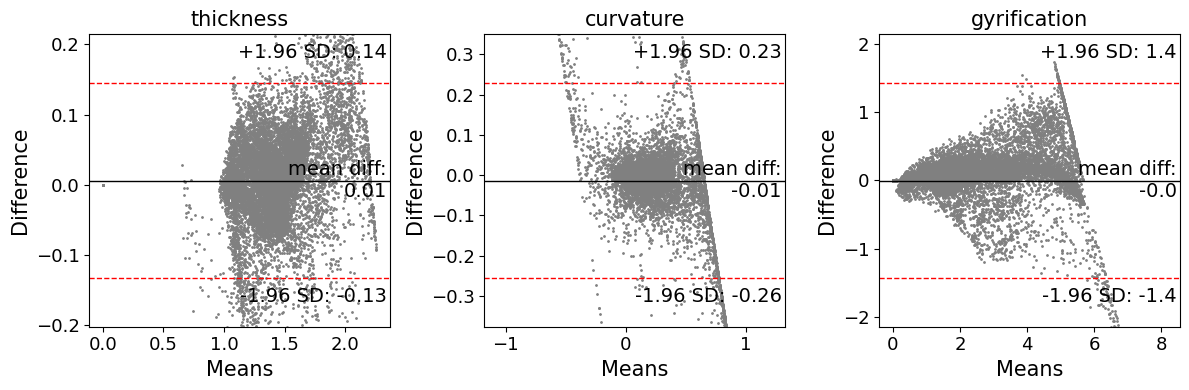


**Supplementary Figure 2: The difference between centile estimates for ipsilateral hippocampal features, without and with adjustment for ICV (C_noICV_-C_ICV_), plotted against patient ICV estimates.** Most patients (95%) had no significant difference in their centile estimates (abs(C_noICV_-C_ICV_)<1.96 standard deviations). Patients with higher ICVs exhibited improved centile estimates (C_noICV_-C_ICV_>0), while those with lower ICVs did worsened (C_noICV_-C_ICV_<0).

**
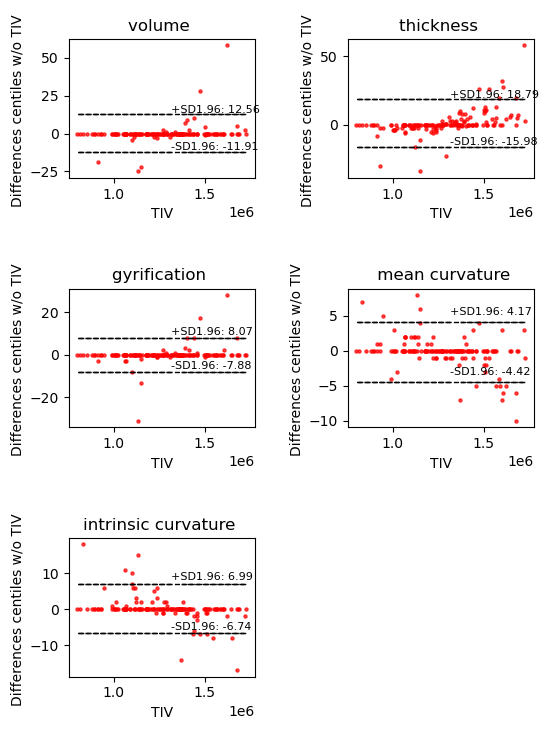
**

**Supplementary Figure 3: Examples of AID-HS reports for two patients with semiology, EEG and imaging features consistent with bilateral HS**. Example 4 additionally underwent intracranial EEG, which supported the diagnosis of no clear unilateral findings. In Examples 3 and 4, four out of five features in both left and right hippocampi were beyond the 5th and 95th percentiles of the healthy population for their age, suggesting bilateral abnormalities. In Example 3, the analysis of asymmetries and the automated classifier predicted a right HS with 87.4% probability, indicating that even though both hippocampi were abnormal compared to the healthy population, the right hippocampus exhibited stronger neuroanatomical abnormalities than the left. On the other hand, Example 4 asymmetries were not lateralising and the classifier predicted no asymmetries with 80.3%, indicating that both the hippocampi appear equally abnormal.


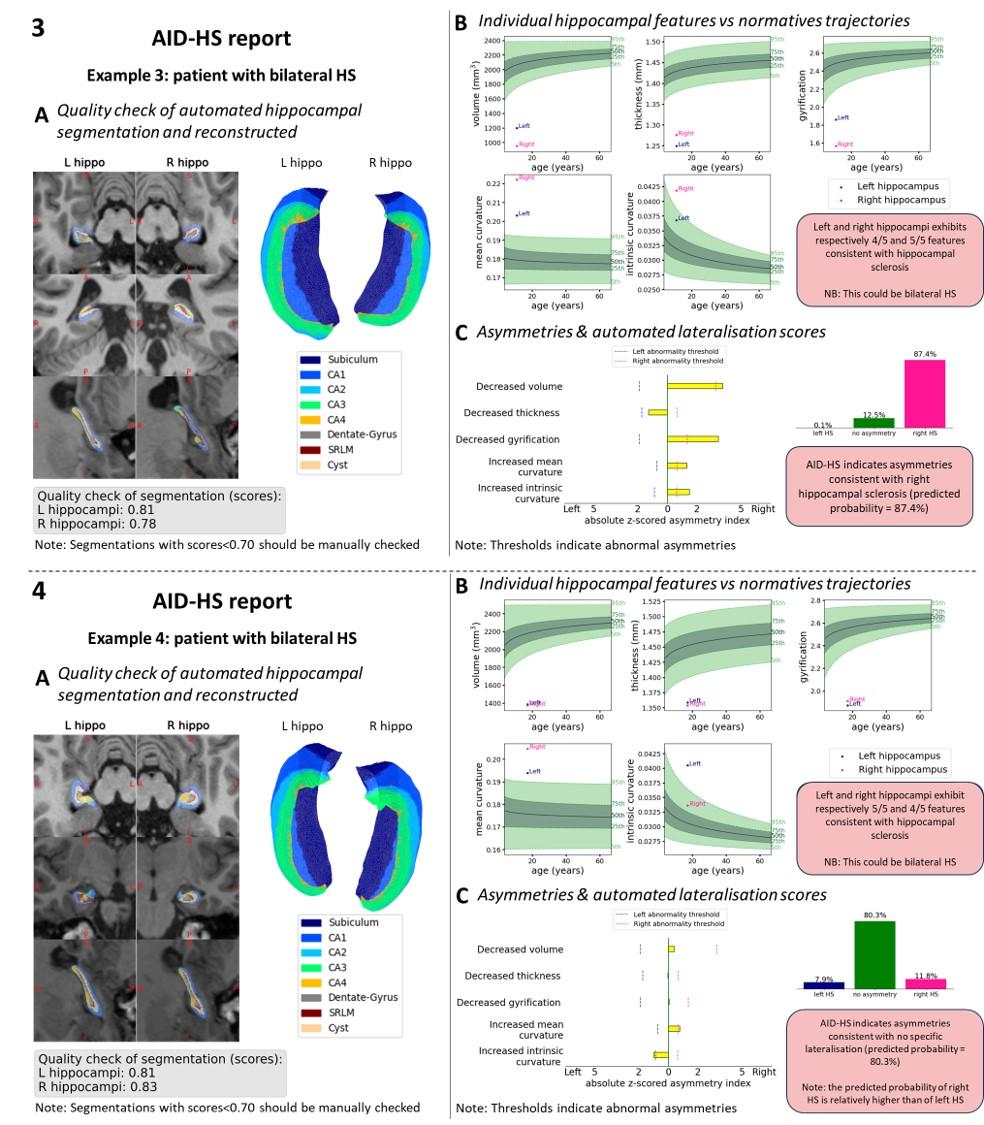


**Supplementary Table 1:** Comparison of AID-HS performances with and without harmonization processing. Evaluation of AID-HS on a subset of the independent test cohort using data preprocessed with and without harmonization using distributed Combat.

|  |  | **Lateralization Sensitivity N=66 patients (n,%)** | **Detection Sensitivity N=66 patients (n, %)** | **Specificity in N=80 controls (n, %)** |
| --- | --- | --- | --- | --- |
| Without harmonization | | 63 (95.5%) | 60 (90.9%) | 79 (98.8%) |
| With harmonization | | 63 (95.5%) | 61 (92.4%) | 77 (96.2%) |

**Supplementary Table 2: Spatial resolution of T1-weighted sequences.** In-plane resolution and slice spacing of the T1w scans from the main cohort and the independent test cohort. Scans were defined as isotropic if the in-plane resolution was within ±0.1mm of the slice spacing.

|  |  | **Main cohort**  **(n=365)** | **Independent test cohort**  **(n=436)** |
| --- | --- | --- | --- |
| In-plane resolution (mm) median [min, max] | | 0.97 [0.41, 1.19] | 0.94 [0.39,1.05] |
| Slice spacing (mm) median [min, max] | | 1.0 [0.9, 2.0] | 1.0 [0.41,3.00] |
| Isotropic resolution | | 258 (90.3%) | 259 (59.4%) |

|  | | | |
| --- | --- | --- | --- |
