# Supplementary material for: Automated and Interpretable Detection of Hippocampal Sclerosis in Temporal Lobe Epilepsy: AID‐HS
Source: Ann Neurol. 2024 Nov 14;97(1):62–75. doi: 10.1002/ana.27089 (PMC11683179; doi:10.1002/ana.27089)
Supplement: Supplementary file 2 — Data S2: MELD HS Study Group. [file ANA-97-62-s002.docx]

**MELD HS Study Group**

**Supplementary Table: MELD HS Study Group:**

| **Author** | **Institution** |
| --- | --- |
| Kai Zhang | Beijing Tiantan Hospital, Beijing, China |
| S. M. Saiful Bari | University Hospital of Wales, Cardiff, UK |
| James Galea | University Hospital of Wales, Cardiff, UK |
| Venkata Sita Priyanka Illapani | Center for Neuroscience, Children's National Hospital, US |
| William D. Gaillard | Center for Neuroscience, Children's National Hospital, US;  George Washington University, US |
| Agustín Ibáñez | Latin America Brain Health Institute (BrainLat), Universidad Adolfo Ibanez, Chile;  Global Brain Health Institute (GBHI), Trinity College Dublin, Ireland |
| Evelyng Faure | Advanced Epilepsy Center, Clínica las Condes, Santiago, Chile;  School of Medicine, Finis Terrae University, Santiago, Chile |
| Manuel Campos | Department of Neurosurgery, Clínica Alemana, Santiago, Chile |
| Mariasavina Severino | Department of Neuroradiology, IRCCS Istituto Giannina Gaslini, Member of the ERN EpiCARE, Genova, Italy |
| Domenico Tortora | Department of Neuroradiology, IRCCS Istituto Giannina Gaslini, Member of the ERN EpiCARE, Genova, Italy |
| Giulia Nobile | Unit of Child Neuropsychiatry, Department of Medical and Surgical Neuroscience and Rehabilitation, IRCCS Istituto Giannina Gaslini, Member of the ERN EpiCARE, Genova, Italy |
| Alessandro Consales | Division of Neurosurgery, IRCCS Istituto Giannina Gaslini, Member of the ERN EpiCARE, Genova, Italy |
| Aswin Chari | Department of Neurosurgery, Great Ormond Street Hospital, London, UK |
| Martin Tisdall | Department of Neurosurgery, Great Ormond Street Hospital, London, UK |
| J. Helen Cross | UCL Great Ormond Street Institute of Child Health, London, UK |
| Callum M. Simpson | School of Computing, Newcastle University, Newcastle upon Tyne, UK |
| Yujiang Wang | School of Computing, Newcastle University, Newcastle upon Tyne, UK |
| Luca De Palma | Neurology, Epilepsy and Movement Disorders, Bambino Gesù Children's Hospital, IRCCS, Member of the ERN EpiCARE, Rome, Italy |
| Alessandro De Benedictis | Neurosurgery Unit, Bambino Gesù Children's Hospital, IRCCS, Member of the ERN EpiCARE, Rome, Italy |
| Lucy Vivash | Department of Neuroscience, The School of Translational Medicine, Monash University, Melbourne, Australia;  Department of Neurology, Alfred Health, Melbourne, Australia |
| Terence J. O'Brien | Department of Neuroscience, The School of Translational Medicine, Monash University, Melbourne, Australia;  Department of Neurology, Alfred Health, Melbourne, Australia |
| Jane De Tisdi | UCL Queen Square Institute of Neurology, London, UK;  National Hospital for Neurology and Neurosurgery, London, UK |
| Marina K.M. Alvim | Department of Neurology, UNICAMP University of Campinas, Campinas, Brazil |
| Fernando Cendes | Department of Neurology, UNICAMP University of Campinas, Campinas, Brazil;  Brazilian Institute of Neuroscience and Neurotechnology, Brazil |
